# Precarious employment and occupational injuries in Sweden between 2006 and 2014: a register-based study

**DOI:** 10.1136/oemed-2022-108604

**Published:** 2022-12-30

**Authors:** Bertina Kreshpaj, David H Wegman, Bo Burstrom, Letitia Davis, Tomas Hemmingsson, Carin Håkansta, Johanna Jonsson, Gun Johansson, Katarina Kjellberg, Nestor Sanchez Martinez, Nuria Matilla-Santander, Cecilia Orellana, Theo Bodin

**Affiliations:** 1 Unit of Occupational Medicine, Institute of Environmental Medicine, Karolinska Institutet, Stockholm, Sweden; 2 Section of Epidemiology, Department of Public Health, University of Copenhagen, Kobenhavn, Denmark; 3 Department of Public Health, University of Massachusetts Lowell, Lowell, Massachusetts, USA; 4 Department of Public Health, Karolinska Institutet, Stockholm, Sweden; 5 Occupational Health Surveillance Program, Massachusetts Department of Public Health, Boston, Massachusetts, USA; 6 Department of Working Life Science, Karlstad University, Karlstad, Sweden; 7 Stockholms Lans Landsting, Stockholm, Sweden

**Keywords:** occupational health, public health surveillance

## Abstract

**Background:**

Precarious employment (PE) has been suggested as a risk factor for occupational injuries (OIs). However, several issues such as under-reporting and time at risk pose obstacles to obtaining unbiased estimates of risk

**Objective:**

To investigate if PE is a risk factor for OIs in Sweden.

**Methods:**

This register-based study included employed workers aged 18–65, resident in Sweden between 2006 and 2014. PE was operationalised as a multidimensional construct (score) and by its five items (contract insecurity, contractual temporariness, multiple jobs/multiple sectors, income level, collective bargaining agreement). Our outcome was OI in the following year. Pooled ORs for OIs in relation to PE and PE items were calculated by means of multivariate logistic regression models for women and men separately.

**Results:**

Precarious workers were at lower risk of OIs as compared with non-precarious workers among both males and females (OR <1) also when applying weights for under-reporting and adjusting for time at risk (part-time work). Male agencies workers had a higher risk of OIs (OR 1.19, 95% CI 1.15 to 1.23), as did male and female workers in multiple jobs/sectors (OR 1.25, 95% CI 1.23 to 1.28 and OR 1.10, 95% CI 1.07 to 1.13 respectively), and female workers in the low-income groups (OR 1.11, 95% CI 1.09 to 1.12). Low coverage of collective bargaining agreements was associated with a lower risk of OIs for both men and women (OR 0.30, 95% CI 0.29 to 0.31 and OR 0.26, 95% CI 0.24 to 0.27, respectively).

**Conclusions:**

While several mechanisms may explain why precarious workers in Sweden present lower risks of OIs, several dimensions of PE such as temp agency work and multiple job-holding could be important risk factors for OIs and merit further research.

**WHAT IS ALREADY KNOWN ON THIS TOPIC**

- The potential relation between precarious employment and occupational injuries is still poorly understood, with the majority of the studies in the literature providing a cross-sectional picture and accounting only for a single aspect of precarious employment.

**WHAT THIS STUDY ADDS**

- This longitudinal study provided results for multiple aspects of precarious employment being risk factor for occupational injuries. Specifically being a multiple job holder as well as working for an agency.
- When measuring precarious employment as a summative scale approach, precarious workers were not found to be at increased risk of occupational injuries, also when accounting for under-reporting as well as differential time-at-risk.

**HOW THIS STUDY MIGHT AFFECT RESEARCH, PRACTICE OR POLICY**

- This work highlighted that tracking the precarious working population in the labour market, accounting for under-reporting levels, and looking at risks of occupational injuries is feasible.
- Monitoring allows for development of policies and programmes to increase workers’ protection in the labour market and develop targeted health and safety programmes to address root causes of occupational injuries.

## Introduction

Workplace injuries pose a significant human, social and economic burden on the individual and on society, and although occupational injuries (OIs) have been declining in the last decades, they remain a major public health concern. In 2018, Eurostat estimated that there were 3.1 million non-fatal work-place injuries that resulted in at least 4 days of sickness absence per injured worker from work in the EU-27.^1^ According to ILOSTAT, Sweden reported 1094 non-fatal OIs per 100 000 workers in 2016, lower than its neighbouring Nordic countries Denmark and Finland (respectively, 1794 and 1726 per 100 000 workers), but higher than Norway (398 per 100 000 workers).^2^ Like other countries, Sweden is affected by the continuous changes in the nature of work, of the workforce and the workplace, which have increased the difficulty in characterising new occupational hazards that could lead to an increase in OIs or other adverse health outcomes.^3–5^ Some of such changes are caused by an increased labour market flexibility that has impacted the traditional employer–employee relationship leading to non-standard and precarious employment (PE) arrangements. PE is an important social determinant of health, encompassing several dimensions of low-quality employment—for example, employment insecurity, income inadequacy and lack of rights and protection—and PE has been associated with adverse physical and mental health outcomes.^6 7^ Rich evidence shows that precarious workers are more likely to work under unsafe workplace conditions and have more stressful and heavier work tasks, shorter job tenure, larger variation in work experience and less in-job-training and awareness of work hazards.^8–10^ In a recent systematic review by our group, we analysed the relationship between OIs and the following aspects of PE: length of employment, work characteristics, income and labour rights.^11^ Included studies found that employees working in multiple jobs and workers employed by temporary agencies had the highest risk of OIs,^12 13^ while those with temporary contracts showed no association with increased risk of OIs,^14 15^ despite that temporary contracts are related to known OIs risk factors such as young age and short job tenure.^16^ Part-time employment is common among precarious workers which is a potential protective factor as it reduces the time-at-risk and also fatigue from long working hours.

Previous studies have also found that unionisation is associated with a higher risk of OIs, probably mediated through a combination of better reporting systems and awareness in unionised workplaces, higher incentives for reporting as workers’ compensation can be linked to unionisation, and lastly that the most hazardous workplaces are more likely to be unionised.^17 18^ A challenge to surveillance and development of targeted measures to prevent of OIs is posed by under-reporting of workplace injuries, which in Sweden has been found to be consistently higher among precarious workers as compared with workers in standard employment relationships.^19^ In sum, the potential relation between PE on OIs is still poorly understood. A majority of the studies in the literature are cross-sectional and account only for a single aspect of PE, while workers’ injury risk may be influenced by multiple aspects of employment quality. This study is designed to improve understanding because it is longitudinal and attempts to account for more than a single aspect of PE. Also, it attempts to address the issues of under-reporting as well as differential time-at-risk between precarious and non-precarious employees.

### Aim and hypothesis

Our hypothesis is that precariously employed workers are at increased risk of OIs compared with non-precarious workers. Thus, the aim of this register-based study is to investigate longitudinally if precarious workers are at higher risk of OIs compared with non-precarious workers in Sweden. Two approaches were used to operationalise PE: (1) by applying a multidimensional construct using the Swedish Register-based Operationalisation of Precarious Employment (SWE-ROPE) and (2) by analysing its five items (contract insecurity, contractual temporariness, multiple jobs/multiple sectors, income level, collective bargaining agreement) to investigate which items may pose a higher risk of OIs.

## Methods

### Study design and data collection

This repeated prospective register-based study includes the total working population aged 18–65, resident and registered in Sweden between 2006 and 2014. Individuals can contribute to multiple years if they fulfil the inclusion criteria at baseline every year. Baseline is defined as the first year (from 2006 to 2014) in which the inclusion criteria is met. The exposure (PE) is measured at baseline and the outcome (OI) is measured at the following year for estimating the risk, and to further account for reverse causality, estimates are adjusted for OI in the previous year. Individual information about the study population was drawn from two main data sources: (1) the Longitudinal Integration Database for Health Insurance and Labour Market Studies register (LISA), which contains employment and demographic data on all Swedish workers and (2) the Information System on Occupational Injuries (ISA), which is a national OI register held by the Swedish Work Environment Authority.^20^

Exclusion criteria for the study at baseline were: (1) yearly employer-based income of <SEK100, (2) death or emigration; (3) self-employed (to avoid misclassifying such workers as precariously employed) and (4) any unemployment spells during the year. Individuals were linked across data sources and years by Statistics Sweden using an anonymised identification number to replace the unique Swedish personal identification number.

### Study variables

#### Exposure

PE level was constructed on the basis of PE scores and calculated for the year before the outcome in order to minimise reversed causality. Data on each individual’s employment were retrieved from LISA from 2003 to 2013 (last year in which exposure was measured). We used the second version of the SWE-ROPE (2.0) to construct a PE scoring based on the original operationalisation of PE.^21^ Briefly, the SWE-ROPE includes five items covering three dimensions of PE: employment insecurity, income inadequacy and lack of rights and protection.^6^ By summing the scores for each of the five items, we obtained a summative score ranging from −9 to –2. PE was defined as a score <−3. Two comparison groups were created, the ‘middle group’ (−3 to −1) and non-precarious (0–2).

#### Outcome

Information on all reported non-fatal OIs were retrieved from the ISA register using the following Swedish legal definition of OIs: ‘an OI is an injury due to accident(s), which occurred at the workplace or other place where the injured person had been for work. For an event to be counted as an accident, it is required that the course was relatively short and arose in connection with a particular event’.^22^ OIs occurring during transit to/from work were excluded from the study since they can be reported to traffic insurance instead. Fatal injuries were also excluded due to the small number over the study period (<50 per year). Included OIs were categorised as injuries that lead to: (A) no sickness absence from work, (B) between 1 and 3 days of sickness absence, (C) between 4 and 14 days of sickness absence and (D) more than 14 days of sickness absence. We further dichotomised our outcome variable into individuals who had an OI and individuals who did not present an OI, for each year between 2006 and 2014. If an individual reported multiple OIs during the same year, the most severe was kept (ie, the OI that led to most days of sickness absence as these are less frequently under-reported).

#### Confounders

A Directed Acyclic Graph was drawn in order to identify the sufficient set of variables for adjustment.^23^ The final confounders collected yearly from LISA were: (1) sociodemographic characteristics (sex, age, educational level and country of birth); (2) OIs in the previous year (if the individual was injured or not during the antecedent year); (3) a proxy variable for working part time or full time to consider time at risk. Percentage of full-time work (time-at-risk) was operationalised using median salary as proxy and stratifying it by economic sector, region, educational level, age and sex. Then, we divided individual salary by the median in the individuals’ corresponding strata. To validate this, we compared our proxy variable for part-time and full-time earnings against the data on part time and full time reported by Statistics Sweden, which confirmed our estimated part-time salary.^24^

### Statistical analysis

Crude and adjusted OR estimates with 95% CIs were calculated for OIs (being or not being injured the following year) by means of pooled multivariate logistic regression models for the study period 2006–2014 using two approaches with different exposure variables: (1) PE (precarious, ‘middle group’ and non-precarious); (2) each PE item (contract insecurity, contractual temporariness, multiple jobs/sectors, income level, collective bargaining agreement). For both approaches the best category in terms of employment quality of each variable was used as reference group: non-precarious workers, directly employed, individuals in stable employment, individuals with 1–2 jobs in the same sector, highest income, highest collective bargaining coverage, not being injured. All analyses were stratified by sex. Further, we conducted several sensitivity analyses: (1) adjusted and unadjusted repeated cross-sectional models were run using exposure and outcome in the same year; (2) adjusted and unadjusted generalised estimating equation (gee) models were run to account for any possible correlation among individuals present in more years; (3) unadjusted and adjusted models of PE and OIs were run for 2013 where the estimates of the under-reporting were included in the model as weights and were based on a previous publication which estimated under-reporting level of OIs for 2013 in Sweden^19^; (4) an unadjusted and adjusted model of PE and OIs was run stratified by economic sector to test if the results would differ across sectors that are known to have higher incidence of OIs; (5) an unadjusted and adjusted model were run across occupations we have found less likely to under-report OIs (add here the occupations?)^19^ and (6) an unadjusted and adjusted model was run by injury severity (categorised as injuries that lead to: (A) no sickness absence from work, (B) 1–3 days of sickness absence, (C) 4–14 days of sickness absence and (D) >14 days of sickness absence). Data management and statistical analysis were conducted using the SAS V.9.4.

## Results

### Sociodemographic characteristics by precarious level and OIs

This study includes 4 794 584 unique individuals during the whole study period.

Table 1 displays the sociodemographic characteristics by precarious level for all individuals at their first appearance in the cohort (ie, the first year where they fulfil the inclusion criteria). Overall, there is a similar proportion of men and women; the majority of individuals are in the mid-age categories (25–34 and 35–54); a higher educational level is found in the non-precarious group, while a higher proportion of non-Swedish workers is found in the precarious and ‘middle group’ groups. When it comes to the ownership sector, the highest proportion of precarious workers belong to the private sector. Table 2 shows OIs according to baseline characteristics of the study subjects by PE level and PE items at their first appearance in the cohort. Most individuals in our sample did not experience an OI (approximately 98%). The precarious group had the smallest share of injured individuals. In the first dimension of PE, a higher share of OIs was found among workers employed by an agency, those having stable employment and multiple job holders with 1–2 jobs in 1–2 economic sectors. In the second dimension, individuals with an income of 60%–120% of the median presented a higher proportion of injured workers. In the third dimension, those with the highest probability of CBA coverage, presented the highest share of OIs.

**Table 1:** Baseline sociodemographic characteristics of included study subjects by level of precarious employment at first appearance in the cohort

	Precarious		Middle group		Non-precarious	
	N	%	N	%	N	%
Sex						
Male	309 635	45.4	1 027 990	42.8	1 074 697	62.9
Female	372 221	54.6	1 375 162	57.2	634 879	37.1
Age						
18–24	286 580	42.0	375 484	15.7	78 644	4.60
25–34	174 090	25.6	631 874	26.2	348 094	20.4
35–54	168 887	24.8	1 013 564	42.1	907 268	53.0
55–65	52 299	7.60	382 230	16.0	375 570	22.0
Educational level						
Missing	3976	0.70	12 143	0.50	9039	0.50
Primary school	81 889	12.0	313 181	13.0	169 688	9.90
Secondary school	405 241	59.4	1 319 215	54.9	704 115	41.2
Tertiary education <3 years	99 189	14.5	301 730	12.6	281 994	16.5
Tertiary education ≥3 years	91 561	13.4	456 883	19.0	544 740	31.9
Country of birth						
Missing	2390	0.40	8303	0.40	5297	0.30
Sweden	577 103	84.6	2 050 558	85.3	1 495 700	87.5
Nordic countries	10 282	1.60	59 641	2.50	52 014	3.00
EU-28	36 733	5.30	130 142	5.40	84 038	4.90
Non-EU-28	55 348	8.10	154 508	6.40	72 527	4.30
Ownership sector						
Private	551 037	80.9	1 445 161	60.1	1 067 615	62.4
Public	130 819	19.1	957 991	39.9	641 961	37.6
Total	681 856	100.0	2 403 152	100.0	1 709 576	100.0

EU, European continent including member and non-members of the EU-28 countries (without Nordic countries).

**Table 2:** Occupational injuries according to precarious employment level and precarious employment items of included study subjects at first appearance in the cohort

	No injury		Injured		Total
	N	%	N	%	N
Precarious employment level					
Precarious	674 087	98.9	7764	1.1	681 851
Middle group	2 359 088	98.2	44 060	1.8	2 403 148
Non precarious	1 677 895	98.1	31 690	1.9	1 709 585
Precarious employment items					
Contractual employment insecurity					
Directly employed	4 639 243	98.3	82 031	1.7	4 721 274
Employed by agency	71 827	98.0	1483	2.0	73 310
Contractual temporariness					
Stable	3 217 340	98.1	60 883	1.9	3 278 223
Unstable	1 493 730	98.5	22 631	1.5	1 516 361
Multiple job/sectors					
1–2 jobs in 1–2 sectors	4 259 496	98.2	76 406	1.8	4 335 902
Three or more jobs in 1–2 sectors	201 233	98.6	2860	1.4	204 093
Three or more jobs jobs in 3+ sectors	250 341	98.3	4248	1.7	254 589
Income level					
>200% of median	117 992	99.7	383	0.3	118 375
120%–200% of median	565 850	99.1	5278	0.9	571 128
80%–120% of median	1 848 093	97.9	39 663	2.1	1 887 756
60%–80% of median	1 222 397	97.9	26 066	2.1	1 248 463
<60% of median	956 738	98.7	12 124	1.3	968 862
Collective bargaining agreement					
91%–100% CBA	4 150 375	98.1	80 555	1.9	4 230 930
71%–90% CBA	420 789	99.4	2341	0.6	423 130
≤70 CBA	139 906	99.6	618	0.4	140 524
Total	4 711 070	98.3	83 514	1.7	4 794 584

CBA, collective bargaining agreement; CBA, collective bargaining agreement.

### PE and risk of OIs

Table 3 displays the associations between PE level and OIs. Both the crude model (model 1) and adjusted model (model 2) show that precarious workers have a decreased risk of OI compared with non-precarious workers, both among male and female workers (OR<1). The results did not change when weighting for under-reporting or stratifying by occupation, economic sector and injury severity (data not shown).

**Table 3:** Multivariate analysis models of occupational injuries by precarious employment level

	Crude model				Adjusted model			
	Male		Female		Male		Female	
	OR	95% CI	OR	95% CI	aOR	95% CI	aOR	95% CI
Precarious employment								
Non-precarious	ref		ref		ref		ref	
Middle group	0.92	0.91 to 0.93	1.03	1.02 to 1.04	0.8	0.79 to 0.80	0.98	0.98 to 0.99
Precarious	0.79	0.78 to 0.81	0.73	0.72 to 0.75	0.65	0.64 to 0.66	0.77	0.75 to 0.78

Model 1 presents crude pooled ORs. Model 2 presents pooled aOR by age, country of birth, educational level, previous year injury and part time and full time.

aOR, adjusted Odds Ratios.

Table 4 shows the associations between each of the items of PE and OIs stratified by sex. The adjusted model (model 2) show that male workers employed by an agency presented higher risk of OIs than those directly employed (aOR 1.19, 95% CI 1.15 to 1.23) while women employed by agencies presented a lower risk (aOR 0.80, 95% CI 0.76 to 0.84). Both female and male workers in unstable employment presented lower risk of OIs as compared with those in a stable employment in both crude and adjusted models. In contrast, multiple jobs holders presented higher risk of OIs across both sexes, with men with >3 employers and working in >3 economic sectors having the highest risk (aOR 1.25, 95% CI 1.23 to 1.28), while women presented a higher risk when working in >3 jobs in 2–3 sectors (aOR 1.13, 95% CI 1.11 to 1.16). All income groups were found to be at reduced risk of OIs when compared with individuals earning between 80% and 119% of the median among male workers. For female workers a reduced OI risk was only found in the group earning more than 120% of the median (aOR 0.50, 95% CI 0.49 to 0.50), while the other income groups presented an increased risk instead (aOR 1.11, 95% CI 1.09 to 1.12 and aOR 1.06, 95% CI 1.05 to 1.08 for those earning 60%–79% and <60% of the median, respectively). In the last item, collective bargaining agreement, both female and male workers being covered by less than 90% of CBA were found having lower risk of OIs then those being covered 91%–100%.

**Table 4:** Multivariate analysis models of occupational injuries by dimensions of precarious employment

Precarious employment items		Model 1				Model 2			
		Male		Female		Male		Female	
		OR	95% CI	OR	95% CI	aOR	95% CI	aOR	95% CI
Dimension 1	Contractual employment insecurity								
	Directly employed	ref		ref		ref		ref	
	Agency employed	1.25	1.21 to 1.29	0.79	0.75 to 0.83	1.19	1.15 to 1.23	0.8	0.76 to 0.84
	Contractual temporariness								
	Stable	ref		ref		ref		ref	
	Unstable	0.9	0.89 to 0.91	0.8	0.79 to 0.81	0.91	0.9 to 0.92	0.86	0.85 to 0.87
	Multiple job/sectors								
	1–2 employers in 1–2 sectors	ref		ref		ref	ref		ref
	Three or more employers in 1–2 sectors	1.12	1.09 to 1.14	1.17	1.15 to 1.19	1.15	1.12 to 1.17	1.13	1.11 to 1.16
	Three or more employers in 3+ sectors	1.2	1.17 to 1.22	1.1	1.08 to 1.13	1.25	1.23 to 1.28	1.1	1.07 to 1.13
Dimension 2	Income level								
	80%–119% of the median	ref		ref		ref		ref	
	>120% of the median	0.46	0.45 to 0.46	0.45	0.44 to 0.45	0.63	0.62 to 0.63	0.5	0.49 to 0.5
	60%–79% of the median	0.89	0.88 to 0.92	1.13	1.12 to 1.14	0.8	0.79 to 0.81	1.11	1.09 to 1.12
	<60% of the median	0.66	0.64 to 0.67	0.89	0.87 to 0.89	0.68	0.67 to 0.7	1.06	1.05 to 1.08
Dimension 3	Collective bargaining agreement								
	91%–100% CBA	ref		ref		ref		ref	
	71%–90% CBA	0.38	0.37 to 0.39	0.27	0.26 to 0.28	0.38	0.37 to 0.39	0.27	0.27 to 0.28
	<=70% CBA	0.3	0.28 to 0.31	0.26	0.24 to 0.27	0.3	0.29 to 0.31	0.26	0.24 to 0.27

Model 1 presents crude pooled ORs, while model 2 presents adjusted pooled OR by age, country of birth, educational level, previous year injury and part time and full time.

AOR, adjusted OR; CBA, collective bargaining agreement.

## Discussion

This study investigated PE as a risk factor for OIs in Sweden between 2006 and 2014. Contradictory to our main hypothesis, individuals in PE were at lower risk of OIs compared with non-precarious employees. Our subanalysis of the PE-items (contract insecurity, contractual temporariness, multiple jobs/sectors, income level, CBA) highlights both the complexity of PE and potential reasons for our unexpected results. In the subanalysis, we found that workers employed by an agency, individuals having three jobs in more than one economic sector, and women with lower salaries were at higher risk of OIs.

At first, we believed that the results were likely biased by under-reporting. In a recent published study of our group, under-reporting of OIs was found to be 50% higher among precariously employed workers as compared with workers in standard employment relationship, where the higher the precarious level, the higher the under-reporting.^19^ This potential differential misclassification of the outcome could partially explain why we do not find an increased risk of OIs for precarious workers. Nevertheless, the results changed only marginally when weights were applied for under-reporting. Time-at-risk was also considered as a possible source of bias, but results remained stable also when adjusting for part-time work.

There are several other possible explanations for the reduced risk of injuries among precarious employees, partly revealed in the item-by-item analysis. In that analysis, our results align with the existing literature which finds an increased risk of OIs for individuals employed by an agency.^13 25 26^ Agency workers are often assigned less desirable and more hazardous working tasks, to shift work and a higher risk of working-task mismatch.^27–29^ Previous studies have also shown an association between OI and performing multiple jobs.^12 14^ Having multiple jobs in the same year is also likely to be a good proxy for very short employment tenure. Short employment tenure is a known risk factor for OIs, and previous studies have indicated that the risk of OI is higher during the first 6 months on the job. In our study, ‘unstable employment’ is defined as having the same employer for less than 3 years, which may unfortunately be a too heterogeneous group with regard to tenure. Another factor to consider is the fact that most of the workers may remain in the same occupation but not necessarily the same job. We did not analyse ‘occupational tenure’ hence our results could be biased by not taking accumulation of occupational experience over time into account. Newly hired employees are less familiar with working duties, thus placing them at higher risk of OIs, but if the worker is able to accumulate job-specific experience in previous jobs with similar working tasks, even though they find themselves in an unstable employment the risk of OIs lowers over time.^30 31^ Giraudo *et al* 32 followed prospectively the evolution of young people careers over the first 6 years after entering the labour market, and they show a protective role played by sector-specific experience gained in previous jobs with similar tasks, confirming a protective effect of accumulated job experience.^32^ Previous studies have shown contradicting results regarding the association between temporary contracts and OIs. In this study, the association was negative. The suggested explanation that our ‘unstable employment’ item has a cut-off of 3 years at the same main employer, which from an OI perspective is irrelevant.

Both male and female workers with a high income showed reduced OI risk while men with lower incomes presented lower risk of OIs while women were at higher risk. In general, low-income earners may tend not to report an injury in order to not lose their job and not lose further earnings while being off work.^19^ However, reasons behind our findings of a differential risk for men and women in this group of low earners are unknown.

Finally, our findings show that workers with a lower coverage of CBA had a much lower risk of OIs. This is not surprising, as the only other two studies that have tested this hypothesis (we are aware of) have shown similar results.^17 18^ With the increase of PE in the last decades, unions have seen their grip on the labour market weaken over time and trade unions are witnessing a limited membership among precarious workers.^33 34^ Some argue that unionised workers are less likely to suffer injuries because unions play a central role in the collective bargaining, promoting education about health and safety practices and arguing for reducing hazardous tasks.^35^ Nevertheless, the few studies that exist suggest that unionised workers are more likely to have OIs and this is driven primarily by the fact that very hazardous workplaces are more likely to unionised. Such hazards have motivated workers to organise in unions in order to protect themselves. Also, unionised and hazardous workplaces where injuries occur more frequently have better reporting systems in place (eg, the police force and paper mill workers in Sweden). Others have also found that unionised workers are more likely to report OIs without fear of reprisal, as compared with non-unionised workers.^36 37^

In summary, the results from single items were more or less as expected. However, the directions of the associations were not the same (both negative and positive). In light of that, it is far from surprising that the multidimensional PE score was not positively associated to the risk of OI.

### Strength and limitations

Being able to construct our exposure and outcome using data from the Swedish registers is a strength in this study for multiple reasons. The high quality and coverage of the national register allows us to include and follow most of the working population in Sweden over time. With the rich and complete information and low missing values, registers also allowed us to construct detailed measurements for both exposure and outcome. Being able to adopt two different approaches in our analysis, using a multidimensional construct for PE and exploring its specific items, provides both a holistic approach and a more in-depth perspective when assessing the precarious level of workers and its potential effect on OI risk. Some methodological limitations must be considered in relation to our study. In our construct of the PE score, we did not have information of working hours for each employee, thus, we tried to account for time at risk by creating a proxy for part-time and full-time employment. Even though such variable was validated through comparison with another statistical source, potential misclassification cannot be ruled out. Furthermore, we were not able to account for job tenure, an important and established factor linked to the risk of OIs. Since employment instability is a major feature of PE, it may be beneficial to collect specific information of how long individuals are hired by a specific employer, and/or working the same job tasks, instead of having yearly information of total number of employments. This would facilitate a better understanding of the role of unstable employment and job tenure across occupations and economic sectors. Finally, we recognise that the need to exclude the self-employed s and those with employment absences limit the generalisability of our finding and the lack of a robust measure for time at risk for those without full-time employment is a study limitation.

## Conclusions

The relation between employment arrangements and work environment is not as obvious as one might think. Some studies indicate that PE is associated with boring and passive rather than hazardous work. In this study, we found, that multiple job-holding and employment through an agency was associated to an increased risk of OI. Although this issue might merit some further research, it is reasonable to assume that the explanation lies in well-known risk factors such as short tenure, lack of safety training and in the case of multiple job-holding, fatigue from inadequate recovery between shifts. Nevertheless, this work highlighted that tracking the precarious working population in the labour market, accounting for under-reporting levels, and looking at risks of OIs is feasible. Monitoring how PE and OIs develop over the life course allow for development of policies and programmes to increase workers’ protection in the labour market and develop targeted health and safety programmes to address root causes of OIs. Thus, longitudinal studies are needed and are encouraged to validate these measurements and operationalisations and to increase international understanding and comparability of these phenomena.

## Data Availability

Data can be obtained through acquisition from Swedish registers. The data collection process is described in the method section of this article. Details from the analysis can be obtained from the corresponding author on request.
